# Examining circadian rhythm dysregulation using actigraphy among treatment-seeking individuals with alcohol use disorder

**DOI:** 10.3389/fnins.2026.1803129

**Published:** 2026-05-01

**Authors:** Gwenyth R. Wallen, Alexandria N. Hughes, Anna E. Roberts, Ralph Thadeus S. Tuason, Ayaan Ahmed, Katherine A. Maki, Jennifer J. Barb

**Affiliations:** Translational Biobehavioral and Health Promotion Branch, Clinical Center, National Institutes of Health, Bethesda, MD, United States

**Keywords:** alcohol use disorder, anxiety, circadian dysregulation, depression, relapse, sleep regularity

## Abstract

**Background:**

Identifying factors predictive of relapse in patients with alcohol use disorder (AUD) following a period of abstinence and/or treatment is essential to discover effective treatment plans for this disease. Previous evidence found that individuals with AUD who relapsed had lower sleep regularity scores than those who did not relapse. This analysis aimed to extend previous work to explore the relationship between circadian rhythms and relapse.

**Methods:**

Treatment-seeking individuals with AUD (*n* = 126) were admitted to an inpatient treatment program for approximately 28 days and, upon discharge, wore Philips Respironics Actiwatches® for the subsequent 4 weeks during an unprotected environment. A subset of these participants wore the devices prior to discharge for up to 7 days inpatient (*n* = 36). Relapse status was assigned if a participant consumed any alcohol during the outpatient period of data collection. Inpatient and outpatient circadian rhythm nonparametric statistics were calculated for all participants including weekly interdaily stability (IS) and intradaily variability (IV), and daily most active 10 h (M10), least active 5 h of the day (L5), relative amplitude (RA), and the wake time. Linear and logistic generalized mixed models were fitted to estimate the effect of discharge on circadian rhythms, the effect of preceding circadian rhythms on the probability of relapse, and the effect of relapse on circadian rhythms. All analyses accounted for within-patient repeated measurements.

**Results:**

The final cohort size was *n* = 103 for the outpatient subset and *n* = 36 for the inpatient subset after actigraphy data filtering. Participants were 48.6 ± 11.3 years of age, and 32% were female. A total of 26 (25.2%) participants relapsed. There were significant decreases in IV and L5 and increases in M10, RA, and wake time between inpatient and outpatient settings, whereas IS did not substantially differ between the two settings. Following relapse, there was a moderate decrease in hourly IV and an increase in (later) wake time of ~1 h; no other circadian variables were significantly predictive of relapse.

**Conclusion:**

Overall, circadian rhythms shifted after discharge but were not predictive of relapse. Instead, relapse was followed by a delayed average wake time and a moderate reduction of daily activity patterns. These results highlight the potential value of monitoring circadian changes as indicators of relapse occurrence rather than relapse risk.

## Introduction

Alcohol use disorder (AUD) is defined as “a problematic pattern of alcohol use leading to clinically significant impairment or distress in an individual” (American Psychiatric Association, 2022). Individuals with AUD are preoccupied with an intense desire to drink alcohol and devote substantial time to obtaining and consuming alcohol. This pattern frequently impairs activities of daily living and increases the risk of developing physical and mental comorbidities (American Psychiatric Association, 2022). According to the Substance Abuse and Mental Health Services Administration (2023), National Survey on Drug Use and Health, reported in the 2023 Annual National Report, an estimated 29.5 million individuals aged 12 years and older met criteria for AUD, with the highest prevalence observed among young adults aged 18–25 years (5.7 million) (Substance Abuse and Mental Health Services Administration, 2023). The economic impact of AUD in the United States in 2010 is estimated at $250 billion (Sacks et al., 2015). Annually, excessive alcohol use is attributed to an estimated 178,000 deaths in the United States and 3 million deaths globally (Centers for Disease Control and Prevention, 2024; World Health Organization, 2018).

Sleep quality and circadian rhythms are closely associated with AUD, with a bidirectional relationship between circadian disruption and alcohol use (Meyrel et al., 2019). However, the extent to which sleep and circadian disturbances contribute to relapse over time remains unclear, and the mechanisms linking sleep and circadian characteristics to alcohol use and misuse are not well understood (Burgess et al., 2024). A 2015 systematic review by Schellekens et al. found only one study evaluating phase markers between individuals with AUD and controls using actigraphy, where they observed no differences between wake-up time and bedtime between the two groups (Meyrel et al., 2019; Schellekens et al., 2015; Boness et al., 2022).

Individuals with AUD often have accompanying comorbidities, including but not limited to depression, anxiety, and post-traumatic stress disorder, that contribute to overall sleep quality (Wallen et al., 2019). A previous study examined objective circadian sleep–wake rhythms in substance use disorder (SUD) patients with major depressive disorder (MDD) and those without MDD comorbidities in ambulatory and therapeutic settings and found higher circadian rhythmicity in SUD patients and therapeutic settings when compared to SUD-MDD and ambulatory settings (Serrano-Serrano et al., 2021; Antúnez et al., 2016). A recent study of males with schizophrenia and accompanying substance use disorder showed that schizophrenic patients and those with SUD experienced longer sleep with a delay in wake-up time, while the SUD-alone patients slept less and showed the highest daily activation and stability even when compared to healthy controls (Adan et al., 2024).

Wearable devices with actigraphy are commonly used to measure activity and sleep behaviors. Actigraphy can provide clinicians with a better understanding of the motor activity and sleep patterns or disruptions that may be present in individuals with AUD. Actigraphy data have been used to characterize the effects of alcohol on sleep and activity (Boness et al., 2022), as well as the circadian rhythms and sleep quality of patients with AUD (Geoghegan et al., 2012; Brooks et al., 2020a). Even low doses of alcohol impact the quantity and quality of sleep through the reduction of total sleep time, where this reduction in sleep time was associated with increased wakefulness during the second half of the night and increased daytime fatigue (Geoghegan et al., 2012).

Insomnia is prevalent in AUD populations, with estimates ranging from 36 to 91%, compared to 10% of the general population (He et al., 2019). Alcohol consumption and intoxication increase sleep fragmentation and reduce deep or restorative sleep (Koob and Colrain, 2019). Sleep regularity may be as important to overall health as sleep duration and is associated with chronic conditions including cardiovascular disease, diabetes, mental health disorders, metabolic abnormalities, and even disruption of the gut microbiome, which could lead to other conditions (Huang et al., 2020; Fritz et al., 2020; Huang and Redline, 2019; Maki et al., 2025; Maki et al., 2026a; Maki et al., 2026b).

The Sleep Regularity Index (SRI) is a measure of sleep behaviors that applies an index to an individual’s sleep–wake consistency over a certain period. In treatment-seeking inpatients with AUD, sleep regularity (increased SRI) occurred during the first 3 weeks of treatment relative to admission (Brooks et al., 2020a), and a follow-up study demonstrated that lower SRI scores were associated with an increased rate of relapse once they are released into their typical, non-protected environment (Barb et al., 2022).

Circadian rhythms are defined as the regulation of cycles of alertness and sleep by an approximately 24-h internal clock. Circadian rhythms can be estimated or objectively quantified using a variety of methods, with the use of actigraphy data collected by a wrist- or hip-worn accelerometer as the most common. Daily circadian rhythm patterns are calculated using variables including interdaily stability (IS) and intradaily variability (IV) to measure usual daily rhythms and intrapersonal deviation across time when longitudinal studies are employed (Witting et al., 1990). Established associations between circadian-related factors and alcohol consumption suggest that maintenance or disruption of circadian rhythms may represent one pathway through which sleep regulation is associated with alcohol-associated patient outcomes. For example, chronotype has been associated with rates of alcohol consumption, and alcohol intake decreases salivary melatonin levels, increases nocturnal body temperature, and may result in a delayed onset of melatonin secretion with increased substance use severity (He et al., 2019; Danel et al., 2001; Meyrel et al., 2019)_._

Therefore, this study evaluated circadian rhythm characteristics in participants with AUD using actigraphy-derived metrics and wake time and assessed whether these characteristics are associated with relapse status following inpatient treatment for AUD.

## Materials and methods

### Study design and participant setting

This analysis aimed to extend previous work and to assess how circadian rhythms are affected between inpatient and outpatient settings and before and after relapse among treatment-seeking individuals with AUD. Participants (*n* = 126) wore actigraphy devices (Philips Respironics Actiwatches^®^; “Actiwatches”) for up to 4 weeks following discharge from the inpatient treatment program at the National Institutes of Health (NIH) Clinical Center. Each participant provided self-reported daily sleep diaries and a daily account of any alcohol intake. A subset of individuals (*n* = 36) wore the devices for approximately 4–7 days prior to discharge, which allowed for a secondary comparison of circadian behaviors during and after discharge from inpatient treatment.

### Study population and clinical data collection

All participants in this study were diagnosed with AUD based on the Structured Clinical Interview for the Diagnostic and Statistical Manual of Mental Disorders (DSM-IV and DSM-5) (First et al., 2002; First et al., 2016; American Psychiatric Association, 2013). All participants met the criteria for AUD, which was the primary diagnosis and reason for inpatient admission, as confirmed by a clinical psychiatrist. Participants were not excluded for comorbid substance use disorders. This circadian analysis builds on previously published work comparing the SRI between participants with AUD who relapsed and those who did not relapse following a 28-day inpatient treatment program to assess the association between SRI and relapse risk (Barb et al., 2022). The primary study from which these data are based recruited inpatients from the NIH Clinical Center and includes actigraphy data from participants who enrolled in the outpatient study (NCT#02181569–“Sleep Disturbance and Relapse in Individuals with Alcohol Dependence: An Exploratory Mixed Methods Study”). All participants were recruited under the National Institute on Alcohol Abuse and Alcoholism (NIAAA) Natural History Protocol (NCT#02231840) and were required to have completed inpatient treatment for at least 14 days. The exclusion criteria were previously described (Danel et al., 2001). This analysis extends previous work but includes additional circadian metrics from actigraphy data using both inpatient and outpatient Actiwatch data from treatment-seeking individuals with AUD (Brooks et al., 2020b). Further details about the study population can be found in previously published manuscripts (Barb et al., 2022; Brooks et al., 2016; Brooks et al., 2020a). Ages for all patients were calculated as the age at the date of consent. Following discharge, participants were followed as outpatients at the NIAAA ambulatory care clinic; however, outpatient treatment engagement was not controlled or systematically recorded. The 28-day follow-up period for the sleep study was observational, with monitoring via actigraphy, sleep diaries, and behavioral assessments. The 28-day follow-up captures early post-discharge risk, not longer-term outcomes.

### Objective sleep data collection using Actiwatches

Sleep actigraphy variables were measured using wrist-worn Actiwatches (worn on the user’s non-dominant wrist) that digitally record gross motor activity. The Actiwatches contained sensors that objectively measure physical activity and sleep variability. Participants were instructed to wear the Philips Respironics Actiwatch Spectrum Plus^®^ throughout the entire data collection window for the outpatient collection time. The data collection window included up to 7 days before patient discharge through 4–6 weeks during the outpatient setting where the participant lived their normal daily life outside of the tightly regulated inpatient treatment environment. A subset of participants wore the watch prior to discharge.

Upon completion of the study window, data from the Actiwatch were downloaded using Philips Actiware software (v. 6.0.9).^1^ Members of the study team performed screening of the data from each participant’s watch and assessed for any malfunctioning devices or data corruption. Data were assessed, and sleep diary information was cross-referenced and validated with the objective data to ensure precise concordance between subjective and objective sleep data. Adjustments were made accordingly if sleep diary responses and actigraphy data are discordant as previously described (Brooks et al., 2020b; some participants needed modifications to their objective data files based on discrepancies found in their sleep diaries). Final cleaned exported files were used for circadian rhythm assessment.

### Data cleaning methods and filtering

**Figure 1:**
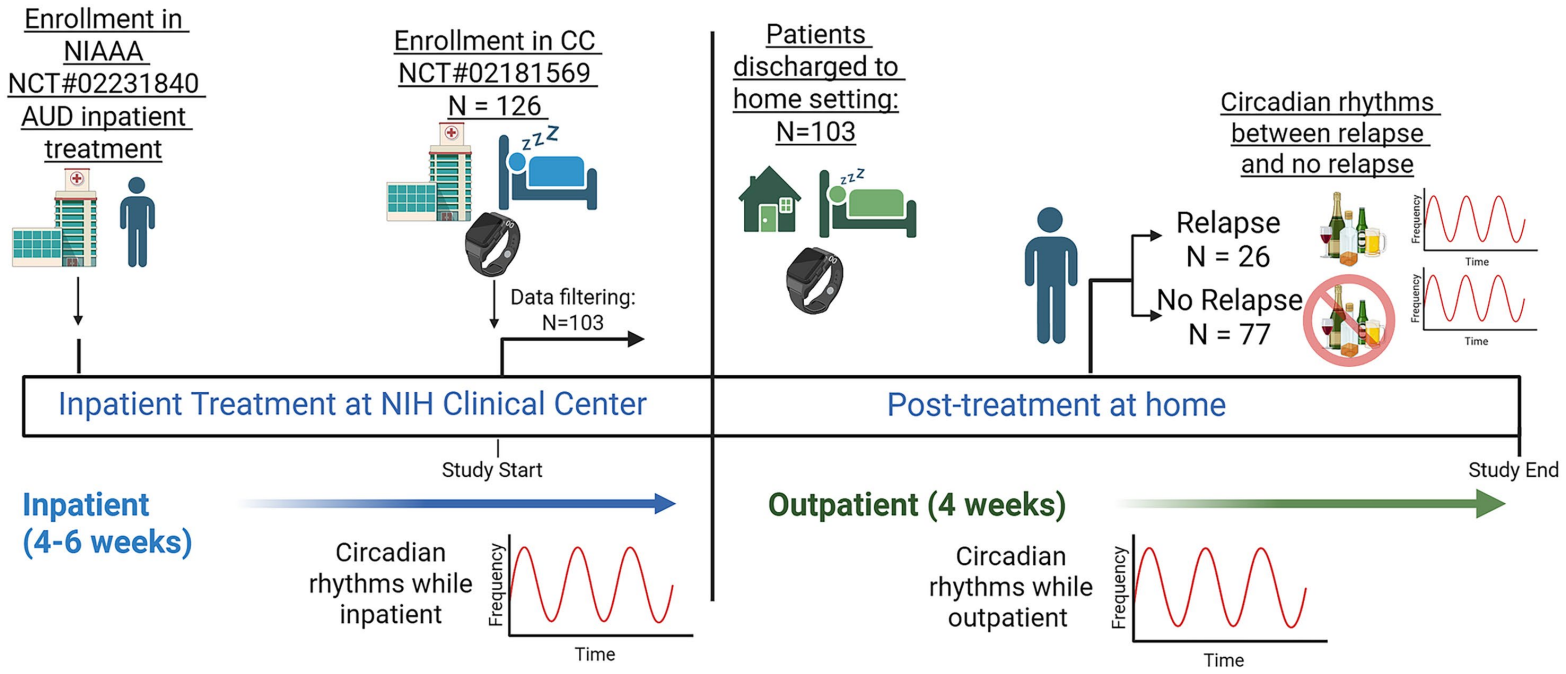
Pictorial graphic of the study design from enrollment into an inpatient treatment program through discharge and the post-treatment follow-up. The patient is first enrolled in the National Institute on Alcohol Abuse and Alcoholism (NIAAA) Alcohol Use Disorder (AUD) inpatient treatment at the National Institutes of Health (NIH) Clinical Center. After inpatient treatment of between 4 and 6 weeks, the patient is enrolled in the Clinical Center (CC) protocol, where circadian rhythms and activity monitoring begin. Following completion of the inpatient program, the patient is then discharged to home settings where they continue circadian and activity monitoring. After 4 weeks of outpatient care, the patient reports their relapse status, and circadian rhythm data was then compared between those that relapsed and those who did not. Figure created in biorender.com.

Actigraphy data were obtained from 126 participants and cleaned using sleep diaries. Five participants did not have sleep diaries and were excluded. We required that within the first 30 days of actigraphy recordings, not more than 30% of the days had more than 6 h of 1-min epochs marked EXCLUDED (indicating non-wear). This led to the exclusion of 18 additional participants (Figure 1; Supplementary Figure S1), resulting in a final cohort of 103 participants who were evaluated in the outpatient setting. Of these, 36 patients had at least 4 days of inpatient data. In the retained cohort, individual days were excluded from the analysis if more than 6 h of 1-min epochs were missing.

### Alcohol- and mental health-related measures

Current and lifetime mood disorder diagnoses were defined by the DSM-IV and DSM-5, where current mood disorders were within the last month, and lifetime mood disorders persisted throughout life. Current and lifetime mood disorders classified by DSM-IV were bipolar I disorder, bipolar II disorder, dysthymic disorder, major depression recurrent, major depression single episode, and mood disorder not otherwise specified. Current mood disorders, as classified by DSM-5, were bipolar I, bipolar II, bipolar other specified, cyclothymic disorder, major depressive disorder, persistent depressive disorder, and other specified depressive disorder. Lifetime mood disorders, as classified by DSM V, were bipolar I, bipolar II, bipolar other specified, major depressive disorder, persistent depressive disorder, and other specified depressive disorder. Furthermore, post-traumatic stress disorder (PTSD) was also classified, which was defined as one experiencing or witnessing a traumatic event, resulting in future mental health effects such as persistent re-experience of the traumatic event, avoidance of stimuli connected with the traumatic event, and increased arousal (Substance Abuse and Mental Health Services Administration, 2016). It is important to note that the diagnostic criteria from the DSM-IV to the DSM-V have been considerably modified, specifically in the consideration of mood disorders. The DSM-V differentiates bipolar disorders from depressive disorders, a major change from the previous version that should be noted (Severus and Bauer, 2013).

The Comprehensive Psychopathological Rating Scale (CPRS) consists of 19 variables that correspond to CPRS-based subscales for affective and anxiety syndromes (Asberg et al., 1978). These subscales are the Montgomery–Åsberg Depression Rating Scale (MADRS) (Montgomery and Åsberg, 1979) and the Brief Scale for Anxiety (BSA) (Tyrer et al., 1984). This measure takes 5–10 min to complete.

The Alcohol Use Disorders Identification Test (AUDIT) is a clinical tool developed to screen adults for hazardous alcohol consumption and was validated for use in six countries—Norway, Australia, Kenya, Bulgaria, Mexico, and the United States (Knight et al., 2003). A score of 8 or more is indicative of hazardous alcohol use (World Health Organization, 2001; World Health Organization, 1989). It is widely used in healthcare organizations to aid in the early detection and early intervention of AUD.

The Alcohol Dependence Scale (ADS) is a 25-item assessment tool with three subscales to assess the severity of alcohol dependence (Mejldal et al., 2019). The ADS has high reliability and validity in categorizing alcohol dependence severity (Doyle and Donovan, 2009). Alcohol dependence levels are divided into four levels based on the total raw score generated: 1–13: low level; 14–21: intermediate level; 22–30: substantial level; and 31–47: severe level of alcohol dependence (Mejldal et al., 2019).

The Clinical Institute Withdrawal Assessment of Alcohol Scale - Revised (CIWA-Ar) is a clinical tool that healthcare providers can use to assess and evaluate interventions administered to patients who are at high risk of or experiencing alcohol withdrawal. The tool consists of 10 items that assess symptoms of alcohol withdrawal—nausea and vomiting, tremor, paroxysmal sweats, anxiety, agitation, tactile disturbances, visual disturbances, auditory disturbances, headache, and clouding of sensorium, in addition to vital signs (Sullivan et al., 1989). It is shown to be especially useful in carrying out a rapid and highly reliable assessment tool to guide alcohol withdrawal treatment management in clinical settings. Withdrawal severity scores are stratified into three categories: minimal to mild withdrawal (<8), moderate withdrawal (8–15), and severe withdrawal (>15) (Foy et al., 1988).

The Penn Alcohol Craving Scale (PACS) is a clinical tool for practitioners to measure alcohol cravings. It is a five-item self-administered instrument that measures frequency, intensity, and duration of thoughts about drinking along with the ability to resist drinking. The final item asks the responder to provide an average rating of his or her craving over the course of the past week. The PACS has excellent internal consistency (Cronbach’s alpha = 0.92). Construct, predictive, and discriminant validity have been established. This assessment takes between 2 and 5 min to complete (Flannery et al., 1999).

The Alcohol Timeline Followback (TLFB) method is a subjective measure of alcohol intake. The TLFB collects drinking information using personal historical events recounted over a fixed time period (Sobell and Sobell, 1992). It is a standard assessment for measuring alcohol drinking patterns and quantification in treatment programs. The number of items corresponds to the number of days of interest, typically 90, which usually takes approximately 30 min to complete. The TLFB has demonstrated high test–retest reliability across multiple populations of drinkers. Content, criterion, and construct validity have been demonstrated in both clinical and general population samples (Sobell and Sobell, 2000). It is extensively used in research and practice (Mason et al., 2013; Connors et al., 1996; Del Boca and Darkes, 2003).

### Relapse assessment

Participants’ relapse status was previously described (Brooks et al., 2020a), but in short, participants were assigned to either the relapse group or the non-relapse group if they reported any alcohol or no alcohol consumption during the time of outpatient data collection, respectively. If a participant consumed any alcohol based on alcohol intake diaries, that participant was placed in the relapse group. Upon diary inspection of the 28-day outpatient study, individuals were dichotomized into either the relapse group or the non-relapse group based on whether they consumed any alcohol during the 28-day outpatient follow-up. Alcohol intake for each day was collected from all participants by a diary of all drinks consumed per day. Total number of drinks (any number of alcoholic drinks), average number of drinks (total number of drinks across the total timeframe divided by the number of days of data collection), and heavy drinking days (four or more drinks for females, five or more drinks for males) were collected during the outpatient data collection timeframe. The days until first relapse were calculated for individuals who consumed any alcohol.

### Actigraphy circadian rhythm analysis

Circadian rhythms were calculated using actigraphy data collected by a wrist-worn accelerometer worn continuously starting within the final week of inpatient treatment and continuing after outpatient discharge. Nonparametric analysis describes circadian rhythms in terms of the variables interdaily stability (IS), intradaily variability (IV), activity of the most active 10 h (M10), and activity of the least active 5 h (L5), which were first described by Witting et al. (1990). These nonparametric measures are well suited to actigraphy data collected under free-living conditions, as they do not assume a sinusoidal waveform and are more robust to irregular or fragmented activity patterns, in contrast to cosinor-based approaches.

Interdaily stability measures the consistency of hourly activity patterns between days, with higher values indicating greater consistency. Intradaily variability reports the activity fragmentation within days by considering the change in activity levels between consecutive minutes or hours. Most active 10 h and L5 summarize the mean or total activity levels during the 10 most and 5 least active hours of each day, respectively, and relative amplitude (RA) represents their difference. In this manuscript, we evaluate circadian behavior on the combined basis of classical nonparametric statistics and algorithmically detected wake time using the Python pyActigraphy package’s implementation of the Crespo algorithm for detecting daily wake time (Crespo et al., 2012).

Actiware files from Philips Respironics^®^ were cleaned using sleep diaries. We performed data processing using both R (4.3.1) and Python (3.10.11) to obtain the circadian metrics interdaily stability (IS), intradaily variability (IV), the mean activity of the most active 10 h of the day (M10), the mean activity of the least 5 active hours of the day (L5), and the daily wake time. IS, IV, M10, and L5 were calculated in R on a weekly (IS and IV) or daily (M10 and L5) basis by the below equations adapted from Witting et al.’s study (1990). Daily wake times were estimated using the Python package pyActigraphy (1.2.1) implementation of the Crespo activity on-time algorithm (Hammad et al., 2021).

For IS, data were first grouped by week and outpatient status, and IS was computed for each week. Data points *X_i_* to *X_N_* are the activity counts for the 1-min epochs within each week. The number of *p* periods is 24 (24 h/day). ${\underline{X}}_{h}\;$ are the hourly means, $\underline{X}\;$ is the overall activity mean. Notably, Witting et al. (1990) used the population variance for the hourly means and individual data points, whereas we used the sample variance for each (note N-1, p-1 terms), a practice also implemented by the pyActigraphy actigraphy analysis package (Hammad et al., 2021).

For IV, data were also grouped by week and outpatient status for each patient to compute weekly IV values. IV was computed hourly; the numerator lag-1 difference is between mean hourly activity counts for successive hours (***X***_***i***_ are hourly means).

M10 and L5 were computed daily for each patient by ranking the total activity counts for each hour and taking the mean of the top 10-h total activity counts (M10) and the mean of the least active 5-h activity counts (L5), as in Witting et al.’s study (1990). RA was calculated by taking the daily difference, M10 – L5.

### Statistical analysis

For each patient, weeks were calculated to count backward from relapse or actigraphy end; for those who relapsed, week 1 spans 7 days pre-relapse, with relapse occurring on the first day of week 0, and for those who did not relapse, week 1 includes the final 7 days of data collection. We required that each week contained at least 4 days of actigraphy data, following the exclusion of individual days exceeding 6 h of missing (non-wear) data. “Weeks” with fewer than 4 days of data did not have weekly statistics (IV and IS) calculated, but daily measures (M10, L5, wake time) were calculated for all valid actigraphy days. To analyze pre−/post-discharge data, a subset of the participants who wore the devices inpatient for at least 4 days were assessed (*N* = 36), including only those individuals with 1 valid inpatient week and including data for the first outpatient week. To estimate the effect of discharge, we fitted linear mixed models with an indicator variable representing outpatient status (inpatient = 0, outpatient = 1) and a random intercept term for repeated measurements within individuals using the lme4 package in R (refer to Supplementary Table S1 for the models). Specifically, we report coefficient estimates for *β* in the models, and *p*-values are from Wald tests for those estimates.

For the assessment of pre- and post-relapse, participants who relapsed were treated as pre-relapse from actigraphy start until the day before relapse and post-relapse, including the relapse day until the end of actigraphy data collection. For individuals who did not relapse, the entire period is used as non-relapsing patient data. To estimate the effect of circadian variables on relapse probability, only pre-relapse period circadian variable values were used. For the binary relapse outcome, logistic mixed models were used to estimate the effect of each circadian variable on the probability of relapse, with a random intercept term for repeated measurements within individuals. The logistic regression models are supplied in Supplementary Table S2.

To estimate the effect of relapse on circadian variables, both pre- and post-relapse data were used. Linear mixed models were adjusted for the effect of discharge and included a random intercept term for repeated measurements within individuals to estimate the effect of relapse (indicator variable; pre-relapse = 0, post-relapse = 1) (refer to Supplementary Table S3). Prior to fitting linear mixed models, we checked assumptions underlying the models. First, the distribution of each outcome parameter was visually inspected to see if any transformations should be considered (e.g., a log transformation to address skewness). Following model fit, we inspected residual plots for patterns or uneven spread indicating deviation from linearity or non-constant variance and the Q–Q plot for comparison of residual distribution with normal distribution quantiles. Limited deviation in the tails of the Q–Q plots was acceptable, with the majority of data points along the line of equality. There were no substantive outliers, and all data points were included in the analyses.

To test whether relapsing and non-relapsing patients differed in circadian parameters over the entire study period, two-tailed T-tests were used, and means, standard deviation (SD), and standardized mean difference (SMD) were reported. For variables with non-symmetric distributions, medians and interquartile range (IQR) were reported.

## Results

### Study population

This study population consisted of a total of 103 treatment-seeking individuals with AUD, of whom 26 experienced a relapse event. Among participants who did not relapse, the median age was 52.6 years; 71.4% (*N* = 55) were male, and inpatient actigraphy was tracked for a median of 2 days (Table 1). Of those who did not relapse, the majority (94.8%; *N* = 73) were not of Hispanic or Latino ethnicity, 26% (*N* = 20) were married, and 22.1% (*N* = 17) were either separated, divorced, or widowed. Finally, 23.4% (*N* = 18) of the non-relapse participants had a mood disorder. Of the 26 individuals who experienced a relapse event, 57.7% (*N* = 15) were male with a median age of 43.52 years. The majority (84.6%, *N* = 22) were not Hispanic or Latino and single (73.1%, *N* = 19). Finally, 34.6% (*N* = 9) of relapsed participants had a mood disorder, and 11.5% (*N* = 3) had PTSD (Table 1). Among participants who relapsed in the outpatient setting, participants had worn the Actiwatch for a median of 1 day during the inpatient setting.

**Table 1:** Participant demographics by relapse status.

Demographic or clinical measure	Did not relapse Median [IQR] or *N* (%)	Relapse Median [IQR] or *N* (%)	*p*	SMD
Total sample size	77	26	–	–
Age	52.61 [42.65, 56.95]	43.52 [36.95, 56.82]	0.126^a^	0.358
Gender (male)	55 (71.4%)	15 (57.7%)	0.292^b^	0.29
Not reported	1 (1.3%)	0 (0.0%)	0.141^c^	0.427
Hispanic or Latino	3 (3.9%)	4 (15.4%)		
Not Hispanic or Latino	73 (94.8%)	22 (84.6%)		
Marital status not specified (missing)	2 (2.6%)	0 (0.0%)	**0.047** ^**c**^	0.732
Married	20 (26.0%)	1 (3.8%)		
Separated/divorced/widowed	17 (22.1%)	6 (23.1%)		
Single	38 (49.4%)	19 (73.1%)		
Days inpatient	2.00 [1.00, 4.00]	1.00 [1.00, 3.00]	0.994^a^	0.059
Daylight saving time change during study window	15 (19.5%)	8 (30.8%)	0.356^b^	0.263
Native American or Alaska Native	1 (1.3%)	0 (0.0%)	0.358^c^	0.445
Asian	2 (2.6%)	1 (3.8%)		
Black/African-American	26 (33.8%)	6 (23.1%)		
Multiracial	2 (2.6%)	1 (3.8%)		
Unknown race	2 (2.6%)	3 (11.5)		
White	44 (57.1)	15 (57.7)		
Mood disorder (DSM-IV/V)	18 (23.4)	9 (34.6)	0.305^c^	0.250
PTSD	9 (11.8)	3 (11.5)	1.0^c^	0.009
BSA, baseline	12.00 [6.00, 16.00]	15.00 [13.00, 17.00]	**0.025** ^**a**^	0.450
MADRS, baseline	15.00 [10.00, 21.00]	19.00 [14.00, 23.00]	0.112^a^	0.342
AUDIT total score	29.00 [24.75, 32.00]	31.00 [26.00, 34.00]	0.102^a^	0.426
ADS score	19.00 [14.75, 24.25]	22.50 [18.25, 29.00]	**0.039** ^**a**^	0.497
CIWA-Ar, baseline	4.00 [2.00, 7.00]	4.00 [1.00, 7.00]	0.766^a^	0.188
Inpatient PACS, baseline	10.00 [5.00, 15.00]	13.00 [5.25, 17.00]	0.274^a^	0.254
Total drinks over 28 days post-discharge	–	16.00 [4.50, 32.25]	–	–
Days until relapse following discharge	–	11.00 [6.25, 13.00]	–	–
TLFB: Total drinks	990.00 [599.50, 1437.00]	1134.00 [729.53, 1586.25]	0.232^a^	0.259
TLFB: Number of drinking days	86.00 [60.00, 90.00]	90.00 [65.75, 90.00]	0.384^a^	0.230
TLFB: Number of non-drinking days	4.00 [0.00, 30.00]	0.00 [0.00, 24.25]	0.384^a^	0.230
TLFB: Average drinks/day	14.74 [9.30, 20.10]	13.69 [10.54, 17.80]	0.991^a^	0.024
TLFB: Heavy drinking days	85.00 [52.00, 90.00]	90.00 [65.00, 90.00]	0.206^a^	0.226

The following tests were run between relapse statuses: ^a^Wilcoxon test, ^b^chi-squared test, and ^c^Fisher’s exact test. PTSD = post-traumatic stress disorder, BSA = Brief Scale for Anxiety, MADRS = Montgomery–Åsberg Depression Rating Scale, AUDIT = Alcohol Use Disorder Identification Test, ADS = Alcohol Dependence Scale, CIWA = Clinical Institute Withdrawal Assessment, PACS = Penn Alcohol Craving Scale, TLFB: Timeline Followback. Bold values indicate *p* < 0.05.

### Mental health- and alcohol-related measures

Participants who relapsed had a significantly higher (*p* = 0.025) baseline anxiety (BSA score) and significantly higher (*p* = 0.039) alcohol severity (ADS score), indicating higher baseline anxiety and alcohol dependence in those who relapsed. Between the relapse/non-relapse groups, there were no significant differences seen in other mental health- and alcohol-related measures (Table 1). Participants who relapsed had a median AUDIT and ADS score of 31 and 22.5, respectively.

### Assessing circadian rhythms pre- and post-discharge for treatment of AUD

To determine the effect of outpatient discharge on circadian rhythms, we restricted our analysis to participants who had at least 4 days of inpatient actigraphy data preceding discharge, which included a subset of 36 participants. A linear mixed-effects model was fitted for each circadian variable for each week (IS and IV) or day (M10, L5, RA, and wake time) as a function of outpatient status and adjusting for within-patient repeated measurements.

**Figure 2:**
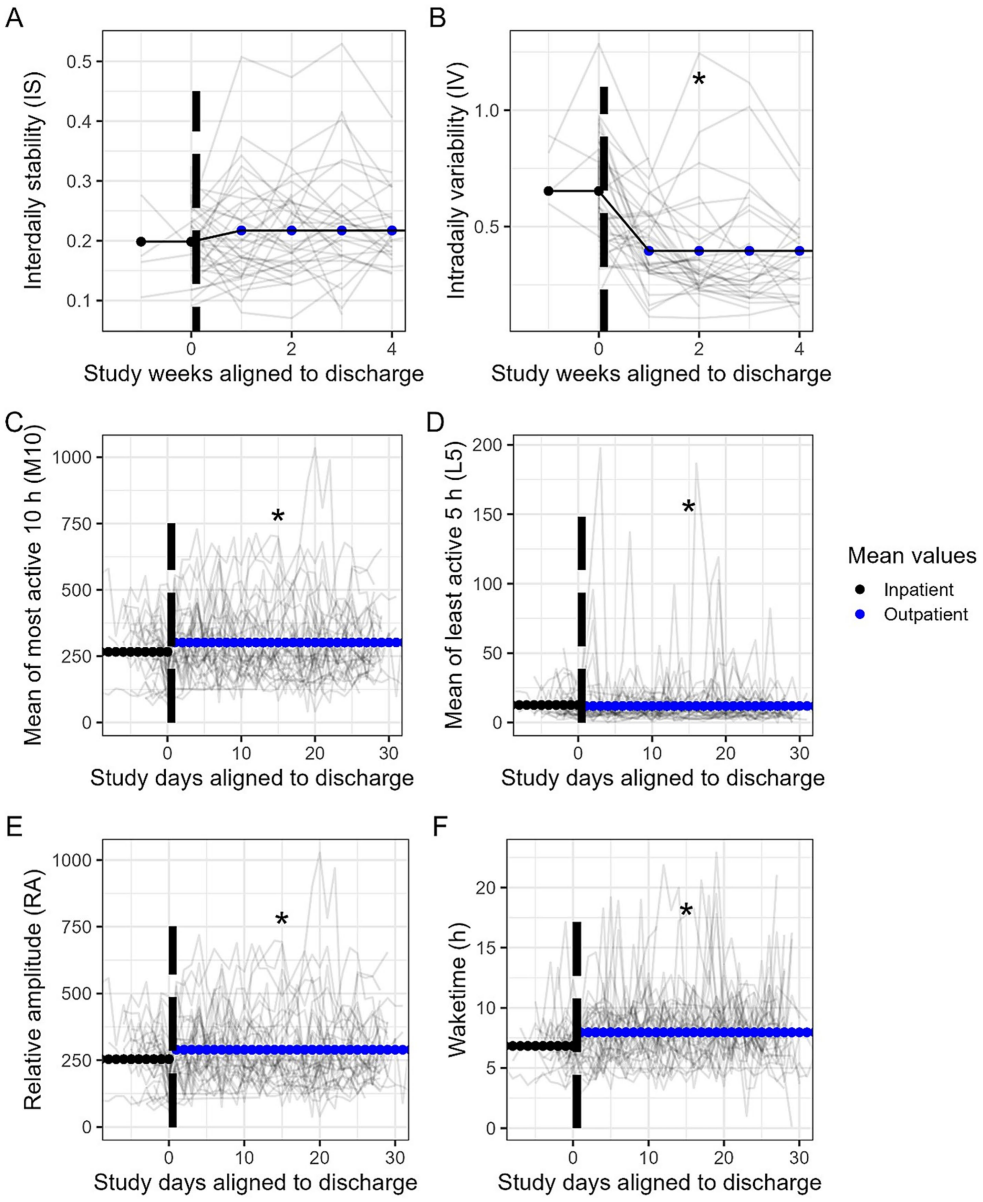
Line plots for circadian variables **(A)** interdaily stability (IS), **(B)** intradaily variability (IV), **(C)** average most active 10 h (M10), **(D)** average least active 5 h (L5), **(E)** relative amplitude (RA), and **(F)** wake time (hours) between inpatient and outpatient settings, represented by study days aligned with discharge. Gray lines represent a specific patient, with the black dotted vertical line representing the start of outpatient timepoints of the study. The average during inpatient is represented by the dotted black line, and the dotted blue line represents the average during outpatient status.

Discharge was associated with widespread changes in these circadian variables. We found that following discharge, there were significant decreases in IV and L5 and increases in M10, RA, and wake time, whereas IS did not substantially change (Table 2; Figure 2). On average, IV decreased by 0.214 units (95% CI: (−0.298, −0.132), *p* < 0.001), M10 increased by 34.97 units (95% CI: (17.53, 52.35), *p* < 0.001), L5 decreased by 3.475 units (95% CI: (−6.32, −0.642), *p* = 0.017), RA increased by 38.32 units (95% CI: (21.06, 55.52), *p* < 0.001), and wake time increased (later) by 3,461 s (0.96 h) (95% CI: (2043.4, 4881.5), *p* < 0.001).

**Table 2:** Regression parameter estimates for the effect of outpatient discharge on circadian variables.

Model response variable *N* = 36	*β* for outpatient	SE (*β*)	df	Test statistic (T)	*p*-value
Interdaily stability (IS)	0.015	0.014	39	1.07	0.290
Intradaily variability (IV)	−0.214	0.042	39	5.10	**<0.001**
Most active 10 h of the day (M10)	34.97	8.868	365	3.94	**<0.001**
Least 5 active hours of the day (L5)	−3.475	1.446	365	−2.40	**0.017**
Relative amplitude (RA)	38.32	8.779	365	4.36	**<0.001**
Wake time (seconds)	3,461	723.2	365	4.79	**<0.001**

Bold values indicate *p* < 0.05.

### Circadian measures by relapse status

Circadian variables were assessed between relapse statuses (Table 3). Of the variables assessed, IS was significantly lower among the participants who experienced a relapse event (*p* = 0.04, SMD = 0.473). Furthermore, the non-relapse group woke up approximately 1 h earlier than the relapse group (*p* = 0.008; SMD = 0.586). No other differences were observed in the reported circadian variables between the two groups.

**Table 3:** Circadian rhythm variables over the entire study period by relapse status.

Circadian rhythm variable	Did not relapse Mean (SD)	Relapsed Mean (SD)	*p*	SMD
Total sample size	77	26		
Intradaily variability (IV)	0.39 (0.14)	0.39 (0.14)	0.982	0.005
Interdaily stability (IS)	0.22 (0.06)	0.19 (0.06)	**0.040**	0.473
Least 5 active hours of the day (L5)	11.59 (5.83)	12.62 (7.69)	0.474	0.151
Most active 10 h of the day (M10)	316.64 (109.94)	293.20 (84.99)	0.324	0.239
Relative amplitude (RA)	305.06 (108.38)	280.58 (85.23)	0.298	0.251
Wake time (HH: MM)	7:44 a.m. (1:29)	8:41 a.m. (1:44)	**0.008**	0.586

Student’s *t*-test; bold indicates *p* < 0.05.

### Circadian dysregulation was not predictive of relapse in this patient cohort

A secondary aim of this study was to investigate whether circadian variables were predictive of relapse during the outpatient setting. During the outpatient period, 26 participants experienced a relapse event, whereas 77 reached the end of the outpatient study period with no relapse (i.e., reported zero alcohol intake). The median number of outpatient days until relapse was 11 (IQR = 4.25–17.75) (mean days until relapse is 10.5 [SD = 6.09], minimum = 1, maximum = 28). Logistic mixed-effects models were fitted with the binary relapse variable (yes or no) as a function of each circadian variable for each week (IS and IV) or day (M10, L5, RA, and wake time) leading up until relapse or study end while adjusting for within-patient repeated measurements. Using these models, we estimated the odds ratio of relapsing for a unit change in each circadian variable (Table 4). We found insufficient evidence for a preceding effect of the circadian variables measured on probability of relapse.

**Table 4:** Logistic regression parameter estimates for the effects of circadian variables on relapse probability.

Circadian predictors	*β*	OR (exp(*β*))	SE(*β*)	Test statistic (Z)	*p*-value
Interdaily stability (IS)	−3.76	0.02	16.4	−0.229	0.819
Intradaily variability (IV)	1.71	5.53	4.30	0.399	0.690
Most active 10 h of the day (M10)	−0.0004	1.00	0.008	−0.047	0.962
Least 5 active hours of the day (L5)	0.0081	1.01	0.050	0.163	0.871
Relative amplitude (RA)	−0.00059	0.999	0.009	−0.069	0.945
Wake time (hours)	−0.00147	0.999	0.384	−0.004	0.997

### Assessing the effect of relapse on circadian rhythms

To determine the effect of relapse on circadian rhythms, we fitted linear mixed-effects models for each circadian variable for each week (IS and IV) or day (M10, L5, RA, and wake time) as a function of pre- or post-relapse status, adjusting for the effect of outpatient discharge, and adjusting for within-patient repeated measurements as before.

**Figure 3:**
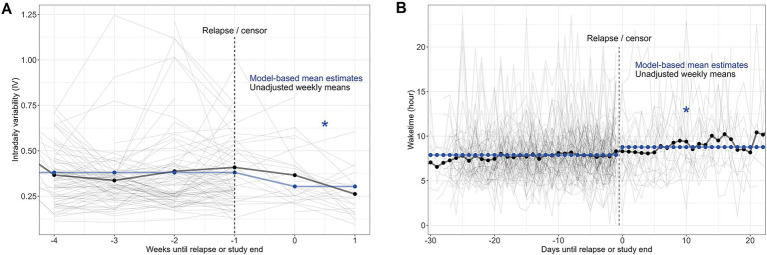
Intradaily variability (IV) by weeks until relapse or the study period ends is visualized within **(A)**, and daily wake times by days until relapse or study end are visualized within **(B)**. For both graphs, each patient is represented by the thin gray traces, where the thicker black lines represent the unadjusted values, and the blue line represents model-fitted values. * indicates *p* < 0.05 for post-relapse.

We found that following relapse, there was a mean 0.08 unit decrease in hourly IV (95% CI: (−0.139, −0.015), *p* = 0.015) and an increased (later) wake time of 3,190 s (0.89 h) (95% CI: (1643.3, 4739.8), *p* < 0.001), whereas other circadian rhythm statistics remained largely unchanged (Table 5; Figure 3).

**Table 5:** Regression parameter estimates for the adjusted effect of relapse on circadian variables.

Model response variable	*β* for relapse	SE(*β*)	df	Test statistic (T)	*p*-value
Interdaily stability (IS)	−0.021	0.011	321	−1.89	0.060
Intradaily variability (IV)	−0.077	0.032	321	−2.44	**0.015**
Most active 10 h of the day (M10)	−15.27	9.512	2,634	−1.61	0.109
Least 5 active hours of the day (L5)	−1.350	1.025	2,634	−1.32	0.188
Relative amplitude (RA)	−13.53	9.371	2,634	−1.44	0.149
Wake time (seconds)	3,190	789.4	2,634	4.04	**<0.001**

Wald test for relapse term from separate linear mixed models for each circadian response variable. Bold values indicate *p* < 0.05.

## Discussion

Sleep disturbances have been established in the literature as a key player in the association between AUD and relapse based on the persistence of these disturbances for weeks into abstinence (Currie et al., 2003). In our previous work with this patient population, sleep regularity (measured by SRI) was significantly lower in individuals who experienced a relapse event following treatment than in those who remained abstinent following discharge from inpatient treatment. SRI was significantly associated with subjective sleep efficiency and sleep quality in the cohort of patients, but overall, SRI was not significantly associated with objective sleep efficiency (Barb et al., 2022). Although SRI represents a relevant aspect of sleep health and is important when assessing sleep patterns individually, this measure does not assess any other aspect of sleep other than sleep–wake consistency. Although our current study focuses exclusively on actigraphy as a measure of circadian rhythms, previous research by others in the field using physiological markers of circadian function, such as skin temperature, has demonstrated associations between rhythmic robustness and clinical characteristics in substance use populations (Adan et al., 2024: Capella et al., 2018). This further supports the need to evaluate multidimensional circadian metrics. The current study extended our previous work by examining how circadian rhythm dysregulation can vary between inpatient and outpatient settings among individuals with AUD, focusing on the transition from inpatient treatment to outpatient care and on changes associated with relapse. By collecting actigraphy data across these transitions, we characterize how discharge affects circadian rhythms, how pre-discharge rhythms may predict relapse, and how relapse impacts subsequent rhythms. These findings can help inform behavioral interventions aimed at stabilizing circadian rhythms during aftercare, potentially reducing relapse risk. This analysis builds on prior work examining sleep regularity in individuals with AUD, extending our understanding of its role during treatment transitions and relapse vulnerability (Barb et al., 2022; Brooks et al., 2016).

The results of this study demonstrate that discharge from inpatient treatment was associated with changes across several circadian rhythm metrics, a finding that is not unexpected given that the transition from a structured inpatient environment to the home setting can be highly disruptive. Following discharge, there were significant decreases in IV and L5 and increases in M10, RA, and wake time. The reduction in IV and L5 suggests greater day-to-day variability and reduced consolidation of the nocturnal rest period in the home environment, consistent with the loss of externally imposed structure and increased exposure to environmental and social cues that may have challenged circadian regularity. Conversely, increases in M10, RA, and wake time indicate heightened daytime activity, a stronger amplitude of the rest–activity cycle, and a shift toward an earlier or more variable wake time as the individuals likely resumed their daily responsibilities. These circadian changes after discharge support our previous quantitative and qualitative sleep data demonstrating that re-adapting to life following release from inpatient treatment also manifests as a detectable shift in many of the circadian variables that were measured in the current study (Brooks et al., 2016)_._ In previous studies, the presence of sleep disturbances from pre- to post-discharge differed significantly, with significantly more sleep disturbances pre-discharge, whereas the proportion of those with daytime sleepiness did not differ significantly between treatment statuses (Brooks et al., 2016). This pattern is consistent with the current circadian findings, suggesting that while subjective daytime alertness may remain relatively stable, underlying circadian organization undergoes marked re-adjustment following transition back to the post-inpatient treatment environment.

When examining circadian rhythms stratified by relapse status, IS was significantly lower in participants who relapsed than in those who did not relapse, indicating less day-to-day regularity of the rest–activity cycle in the relapse group. Lower IS reflects weaker coupling of the sleep–wake rhythm to external zeitgebers and greater variability in sleep and wake timing, which may reduce sleep-related homeostatic and circadian buffering and increase vulnerability to reward-seeking behaviors. This reinforces our previous work on the outpatient population, showing that lower sleep/circadian regularity is associated with poorer substance use outcomes following treatment (Barb et al., 2022; Currie et al., 2003).

In addition, the observation that wake time in the relapse group was approximately 1 h later on average further supports the presence of circadian disruption, specifically a delayed or less stable timing of the active period, among those who returned to drinking. Experimental and observational studies indicate that alcohol exposure and circadian misalignment can alter the amplitude and timing of rest–activity rhythms, and these alterations have been associated with increased alcohol use and impaired reward regulation (Meyrel et al., 2019; Hasler and Clark, 2013).

Importantly, the relapse group also had higher baseline anxiety and greater alcohol dependence severity measured. These clinical variables are established predictors of relapse and likely interact with sleep/circadian dysregulation to increase relapse risk: anxiety may worsen sleep regularity and drive alcohol use as self-medication, while higher dependence severity may magnify sleep disturbance that persists into early recovery (Schellekens et al., 2015; Hasler et al., 2014; Brower and Perron, 2010). Taken together, our results suggest a model in which preexisting clinical severity (anxiety and dependence) and objective circadian irregularity jointly mark individuals at elevated short-term relapse risk, highlighting potential intervention targets (e.g., stabilizing sleep/wake timing, behavioral chronotherapy, and early anxiety management). Support for sleep- and circadian-related intervention targets has been validated in the literature (Barb et al., 2022; Springer et al., 2025; Türkmen et al., 2025), pointing to the potential of this model to strengthen AUD treatment effectiveness altogether and as a promising future direction.

### Strengths and limitations

Several limitations of this study warrant consideration. This study included both inpatient and outpatient objective actigraphy data. The difference in the sample size between inpatient and outpatient on circadian dysregulation was assessed; the small sample size during inpatient treatment limits the ability to draw conclusive findings. Future research is needed on this topic to thoroughly assess circadian metrics between inpatient and outpatient settings. Second, differences observed between inpatient and outpatient or residential treatment settings may be influenced by the transition period following discharge rather than treatment status alone. The process of moving from a highly structured environment with continuous clinical support to a setting characterized by greater autonomy and fewer external constraints represents a substantial behavioral and psychosocial shift. As such, future studies examining associations between circadian dysregulation and relapse risk should explicitly account for the transition from structured treatment to less structured environments when interpreting circadian- and sleep-related outcomes. Within these stressful conditions, patients are still expected to follow their recovery goals and maintain abstinence while adjusting to familiar or new environments and added real-world pressures. Real-life setting struggles that are endured while transitioning between treatment statuses have been described within individuals with AUD and previously published by our team (Brooks et al., 2016). Additionally, we defined relapse as any alcohol consumption during the follow-up period. While this approach is sensitive to early return to use, it does not distinguish between isolated lapses and more sustained or clinically significant relapse patterns (e.g., heavy drinking). The 28-day follow-up period captures early post-discharge risk but does not reflect longer-term relapse trajectories. Future studies incorporating longer follow-up and more granular measures of relapse severity are warranted. Although AUD was the primary diagnosis, comorbid substance use was not systematically assessed or controlled for. Given the high prevalence of polysubstance use in this population, its potential influence on circadian rhythms cannot be excluded. Finally, marital status was not included as a covariate. Given evidence that social and relational factors may influence relapse risk, future studies should consider incorporating these variables to better understand recovery trajectories.

The findings of the present study highlight several important implications and directions for future research. Given the dynamic changes in circadian rhythms observed during the transition from inpatient to outpatient care, future studies with sufficient power are needed to explicitly compare circadian regulation across treatment settings and to disentangle treatment effects from post-discharge adaptation. In particular, longitudinal studies examining whether circadian rhythm measures that were assessed during the early outpatient period, or even immediately prior to discharge, predict relapse risk at clinically meaningful time points (e.g., 1 week, 2 weeks, and 1 month post-discharge) would provide valuable insights into the temporal relationship between circadian dysregulation and relapse vulnerability. In addition to circadian timing, future studies should also consider the intersection of circadian rhythms with other modifiable health behaviors, including physical activity, dietary patterns, and sleep quality, which may collectively influence recovery trajectories.

## Conclusion

Taken together, our results suggest a model in which preexisting clinical severity (anxiety and alcohol dependence) and objective circadian irregularity jointly mark individuals at elevated short-term relapse risk, highlighting potential intervention targets (e.g., stabilizing sleep/wake timing, behavioral chronotherapy, and early anxiety management). These findings provide preliminary evidence and suggest the need to continue to study possible circadian rhythm disruption that may occur during drinking through abstinence and possible relapse.

## Data Availability

Supporting material is available in the Supplementary files. Code used for the manuscript is available at: https://github.com/hughesan/circadian-aud-2026. Other data is available by request from the corresponding author.

## Footnotes

1
https://www.usa.philips.com/healthcare/sites/actigraphy/solutions/actiware

## Author disclaimer

The contributions of the NIH author(s) were made as part of their official duties as NIH federal employees, are in compliance with agency policy requirements, and are considered Works of the United States Government. However, the findings and conclusions presented in this paper are those of the author(s) and do not necessarily reflect the views of the NIH or the U.S. Department of Health and Human Services.
