# Supplementary material for: Examining circadian rhythm dysregulation using actigraphy among treatment-seeking individuals with alcohol use disorder
Source: Front Neurosci. 2026 May 1;20:1803129. doi: 10.3389/fnins.2026.1803129 (PMC13176302; doi:10.3389/fnins.2026.1803129)
Supplement: Supplementary file 1 [file Data_Sheet_1.DOCX]

**Supplemental Material**

**Figure S1: Heatmap of actigraphy data quality for each day of patient Actiwatch wear.**

**
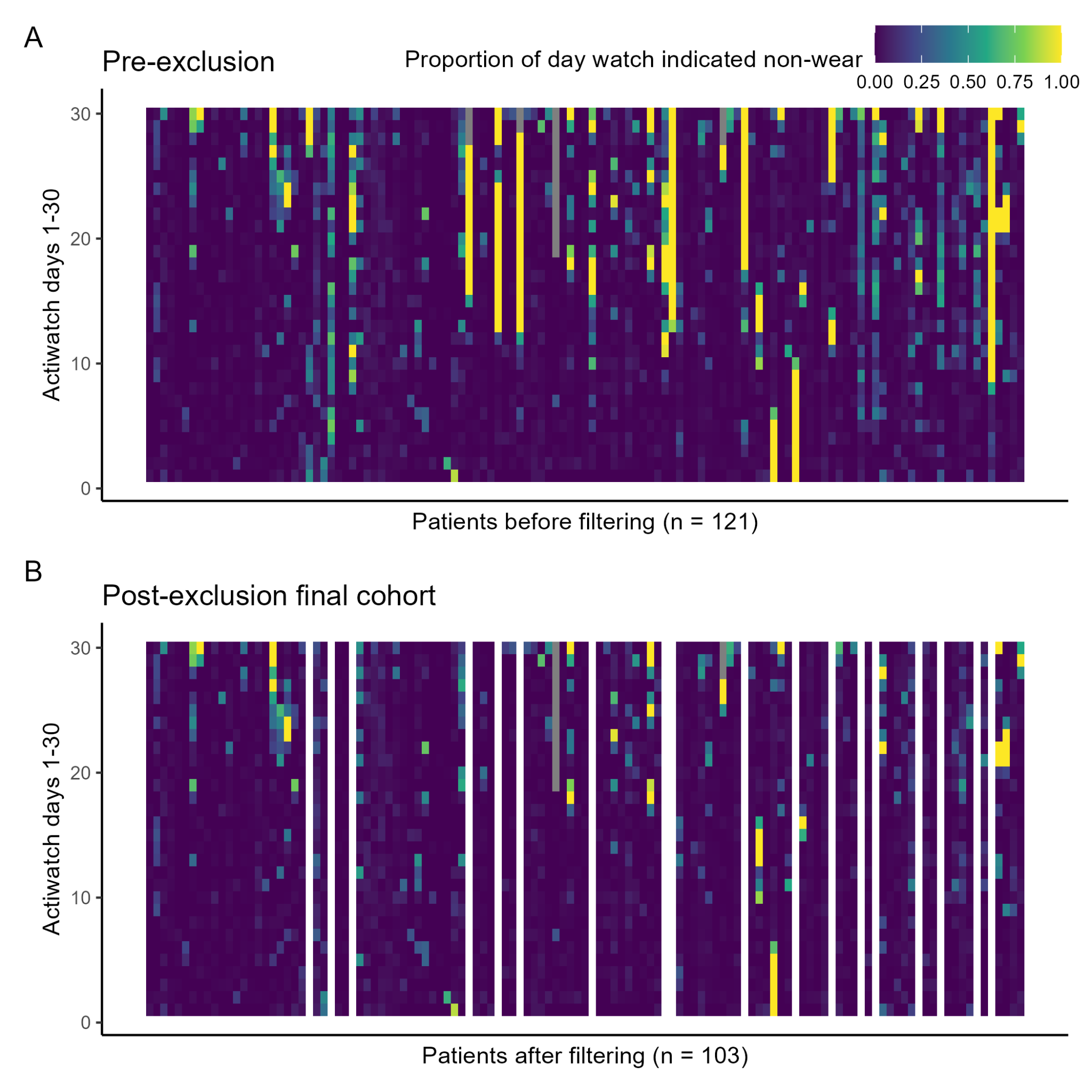
Supplementary Figure 1 Legend.** Heatmaps of actigraphy data quality for each day of patient Actiwatch wear before and after filtering. Each column is 1 patient, and each row is 1 day, with days of actigraphy data collection numbered 1-30 (30 days of wear are shown). The proportion of each day that the watch was not worn is represented by the viridis color scale, with brighter colors indicating days with a low proportion of wear. (A) All patients with actigraphy data cleaned with sleep diaries, and (B) patients after removal of patients with excessive non-wear.

**Table S1: Repeated Measurements Models in R**

| IS_ij = β*outpatient_ij + patient_i + error_ij | lmer(IS ~ outpatient + (1\|PID)) |
| --- | --- |
| IV_ij = β*outpatient_ij + patient_i + error_ij | lmer(IV ~ outpatient + (1\|PID)) |
| M10_ij = β*outpatient_ij + patient_i + error_ij | lmer(M10 ~ outpatient + (1\|PID)) |
| L5_ij = β*outpatient_ij + patient_i + error_ij | lmer(L5 ~ outpatient + (1\|PID)) |
| RA_ij = β*outpatient_ij + patient_i + error_ij | lmer(RA ~ outpatient + (1\|PID)) |
| waketime_ij = β*outpatient_ij + patient_i + error_ij | lmer(waketime ~ outpatient + (1\|PID)) |

Description: A random intercept term for repeated measurements within patients using the lme4 package in R. Specifically, we report coefficient estimates for β in the following models accompanied by their model statements in R

**Table S2: Logistic Mixed Models used in R**

| logit(relapsed_ij) = β*IS_ij + patient_i | glmer(relapsed ~ IS + (1\|PID)) |
| --- | --- |
| logit(relapsed_ij) = β*IV_ij + patient_i | glmer(relapsed ~ IV + (1\|PID)) |
| logit(relapsed_ij) = β*M10_ij + patient_i | glmer(relapsed ~ M10 + (1\|PID)) |
| logit(relapsed_ij) = β*L5_ij + patient_i | glmer(relapsed ~ L5 + (1\|PID)) |
| logit(relapsed_ij) = β*RA_ij + patient_i | glmer(relapsed ~ RA + (1\|PID)) |
| logit(relapsed_ij) = β*waketime_ij + patient_i | glmer(relapsed ~ waketime + (1\|PID)) |

Description: To estimate the effect of circadian variables on relapse probability, only pre-relapse circadian variable values were used. For the binary relapse outcome, logistic mixed models were used to estimate the effect of each circadian variable on probability of relapse, with a random intercept term for repeated measurements within patients.

**Table S3:** **Linear mixed models to estimate the effect of relapse in R**

| IS_ij = β_1*postrelapse_ij + β_2*outpatient_ij + patient_i + error_ij | lmer(IS ~ postrelapse + outpatient + (1\|PID)) |
| --- | --- |
| IV_ij = β_1*postrelapse_ij + β_2*outpatient_ij + patient_i + error_ij | lmer(IV ~ postrelapse + outpatient + (1\|PID)) |
| M10_ij = β_1*postrelapse_ij + β_2*outpatient_ij + patient_i + error_ij | lmer(M10 ~ postrelapse + outpatient + (1\|PID)) |
| L5_ij = β_1*postrelapse_ij + β_2*outpatient_ij + patient_i + error_ij | lmer(L5 ~ postrelapse + outpatient + (1\|PID)) |
| RA_ij = β_1*postrelapse_ij + β_2*outpatient_ij + patient_i + error_ij | lmer(RA ~ postrelapse + outpatient + (1\|PID)) |
| waketime_ij = β_1*postrelapse_ij + β_2*outpatient_ij + patient_i + error_ij | lmer(waketime ~ postrelapse + outpatient + (1\|PID)) |

Description: To estimate the effect of relapse on circadian variables, both pre- and post-relapse data were used. Linear mixed models to estimate the effect of relapse (indicator variable; pre-relapse = 0, post-relapse = 1) were adjusted for the effect of discharge and included a random intercept term for repeated measurements within patients.
